# Protocol development for somatic embryogenesis, SSR markers and genetic modification of *Stipagrostis pennata* (Trin.) De Winter

**DOI:** 10.1186/s13007-021-00768-9

**Published:** 2021-06-30

**Authors:** Masoumeh Asadi-Aghbolaghi, Beata Dedicova, Sonali Sachi Ranade, Kim-Cuong Le, Farzad Sharifzadeh, Mansoor Omidi, Ulrika Egertsdotter

**Affiliations:** 1 Department of Agronomy and Plant Breeding, College of Agriculture and Natural Resources, University of Tehran, 14174 Karaj, Iran; 2 Department of Forest Genetics and Plant Physiology, Umeå Plant Science Centre, Swedish University of Agricultural Sciences, 90183 Umeå, Sweden

**Keywords:** Grass, *Stipagrostis pennata* (Trin.) De Winter, Somatic embryogenesis, Plant regeneration, SSR markers, *Agrobacterium*

## Abstract

**Background:**

*Stipagrostis pennata* (Trin.) De Winter is an important species for fixing sand in shifting and semi-fixed sandy lands, for grazing, and potentially as a source of lignocellulose fibres for pulp and paper industry. The seeds have low viability, which limits uses for revegetation. Somatic embryogenesis offers an alternative method for obtaining large numbers of plants from limited seed sources.

**Results:**

A protocol for plant regeneration from somatic embryos of *S. pennata* was developed. Somatic embryogenesis was induced on Murashige & Skoog (MS) medium supplemented with 3 mg·L^–1^ 2,4-D subsequently shoots were induced on MS medium and supplemented with 5 mg·L^–1^ zeatin riboside. The highest shoots induction was obtained when embryogenic callus derived from mature embryos (96%) in combination with MS filter-sterilized medium was used from Khuzestan location. The genetic stability of regenerated plants was analysed using ten simple sequence repeats (SSR) markers from *S. pennata* which showed no somaclonal variation in regenerated plants from somatic embryos of *S. pennata*. The regenerated plants of *S. pennata* showed genetic stability without any somaclonal variation for the four pairs of primers that gave the expected amplicon sizes. This data seems very reliable as three of the PCR products belonged to the coding region of the genome.

Furthermore, stable expression of GUS was obtained after *Agrobacterium*-mediated transformation using a super binary vector carried by a bacterial strain LBA4404.

**Conclusion:**

To our knowledge, the current work is the first attempt to develop an in vitro protocol for somatic embryogenesis including the SSR marker analyses of regenerated plants, and *Agrobacterium*-mediated transformation of *S. pennata* that can be used for its large-scale production for commercial purposes.

## Background

*Stipagrostis pennata* (Trin.) De Winter belongs to the Poaceae subfamily Aristidoideae that holds only three genera (*Aristida, Stipagrostis,* and *Sartidia*) [1, 2]. *S. pennata* is a three-awned, perennial psammophytic grass common to desert areas in Iran, Afghanistan, Turkmenistan, China, Saudi Arabia, and Iraq [3]. All known *Stipagrostis* species have a C4 photosynthetic pathway which enable them to survive in harsh and hot environments [4]. Furthermore, *S. pennata* has been shown to be able to host a microbiome that allows it to be a pioneer plant on nitrogen-deficient desert soils [5]. It is utilized for fixing sand in shifting and semi-fixed sandy lands, showing a strong tolerance to aridness, wind erosion, and sand embedding [6] and for grazing both in its green and dry stages, which makes this grass species important for landscaping and creating large pastures in arid and light sandy soils [7]. In Tunisia, *Stipagrostis pungens* is cultivated in large quantities as a source of lignocellulose fibres for pulp and paper industry [8]. The flowering and fruiting periods are usually from May until August, and the seeds are easy to cast [9]; however, the seeds show low viability and there is a shortage of plants for revegetation.

Clonal propagation by somatic embryogenesis offers an alternative propagation method with the potential to provide many plants from limited number of seeds. Protocols for somatic embryogenesis has been established for many valuable grass species like napiergrass (*Pennisetum purpureum* Schumach.) [10], reed grass (*Phragites australis* Cav.) [11]. Somatic embryogenesis also offers a platform technology for improving traits by genetic transformation and has been utilized for genetic transformation in millets [12] and switchgrass (*Panicum virgatum* L.) [13]. Furthermore, the model grass species *Brachypodium distachyon* has been frequently utilized for optimization of in vitro protocols and fundamental research [14]. To date, there are however no reports on somatic embryogenesis and genetic transformation in any species within the Aristidoideae subfamily.

Our work is the first attempt to develop an in vitro protocol for somatic embryogenesis of *S. pennata,* as well as testing the embryogenic callus for its competence to be genetically modified via *Agrobacterium*-mediated transformation. For both these technologies, the key question still remains, if the plants regenerated in vitro are genetically stable and they are not exhibiting any somaclonal variation described e.g. by D’Amato [15], Sree Ramulu et al. [16], Linacero et al. [17], Guo et al. [18] and Gao et al. [19]. Somaclonal variations are often observed in monocotyledonous and dicotyledonous species regenerated from different in vitro cultures. This genomic instability can be further analysed and confirmed by using techniques such as flow cytometry or by using molecular markers e.g. random amplified polymorphic DNA (RAPD) markers and simple sequence repeats (SSR) markers.

Microsatellites or simple sequence repeats (SSR) or short tandem repeats (STR) are molecular markers comprised of short repetitive DNA sequences of one to six nucleotides which result from mutations due to DNA polymerase slippage during replication and unequal recombination [20]. SSRs are highly polymorphic and their mutation rate is generally considered to be high (10^−2^ to 10^−5^ per locus per replication) as compared to that of single nucleotide polymorphisms (SNPs), and there are several advantages of SSRs over SNPs, the most important being the cost effectiveness and reliability [21]. SSRs are widely dispersed along the genome and are codominant, and also multi-allelic in nature; therefore SSRs are widely used as molecular markers in population genetics studies in plants which can be obtained by amplification the SSR containing regions by the PCR, once the primers are developed [22]. SSRs were used as molecular markers for analysis of genetic diversity and population structure in several cereal grass species e.g. pearl millet [23], rice [24], wheat [25], maize [26], barley [27], and rye [28]. SSRs have been also used to study the genetic structure in non-cereal grass species which are useful for foraging or paper/pulp industry or are of ecological and economical values such as bamboo [29], reed canary grass [30], guinea grass [31], *Elymus nutans* [32], ryegrass [33], buffalo grass [34], and centipede grass [35]. SSR transferability across species and sub-genera has been demonstrated by earlier studies in several plant genera e.g. *Pinus* [36], *Cereus* [37], *Betula* [38], and *Hibiscus* [39]. With reference to the Poaceae family, cross-species transferability of SSRs has been shown in sugarcane [40, 41], guinea grass [31], ryegrass [33], bamboo [42], and koronivia grass [43]. In plants, genetic changes that occur in mitotically dividing cells lead to somatic mutations, which are frequently caused by series of environmental factors [44]. Stress (biotic/abiotic) can also induce genome instability and somatic mutations in plants [45, 46]. Since the SSRs are highly polymorphic and their mutation rate is generally considered to be high, they can be used to analyse the genome stability, which is a cost effective way [47].

*Agrobacterium*-mediated transformation of monocotyledonous species has been for a long time technically challenging, especially for cereals [48, 49]. It took more than ten years after publishing the first successful *Agrobacterium*-mediated transformation of tobacco by De Block et al. [50] to produce the transgenic plants in crops such as rice [51], and maize [52]. Successful production of transgenic wheat and barley was done even later [53, 54]. Despite the massive success in the area of usage of various transformation techniques and production of transgenic plants, its broad implementation for monocots species e.g. oats [55] and rye [56] still required improvements. Modifications and successful development of the existing transformation protocols including also completely new techniques and approaches [57–61]. Moreover, there exists plant species such as grasses where this technique has not been tested effectively or widely implemented. One such species is *S. pennata,* where lack of reproducible and reliable protocol for plant regeneration via somatic embryogenesis has been a limiting factor for genetic modification. Our research was conducted to consider the effect of different callus induction media for formation of somatic embryos and genetic stability of regenerated in vitro plants.

## Methods

### Plant material and induction of somatic embryogenesis

Mature and immature caryopses of *S. pennata* were collected from field-grown, self-pollinated plants from two locations in Iran (Khuzestan 31° 32′ 11.5″ N lat 49° 03′ 04.3″ E long; South Khorasan—32° 38′ 16.6″ N lat 59° 05′ 15.7″ E long,). The seeds were surface sterilized with 70% ethanol for one min followed with 2% sodium hypochlorite for 10 min and rinsed by sterile Mili Q water three times. Four different explants were tested for callus induction and somatic embryos formation: cut caryopses, mature zygotic embryos, immature zygotic embryos, and leaf bases.

Caryopses after sterilization were transversely cut in halve. Mature and immature embryos (0.5–2.0 mm long) were isolated under a stereomicroscope (Leica E4, Germany) and placed with the scutellum side up on callus induction medium. Leaf bases, we obtained by cutting lower part of young seedlings germinated on MS hormone free medium [62] for a week at 23 °C in the dark. All explants were cultured on MS callus induction medium (Table 1) with pH 5.6–5.8 adjusted before autoclaving or filter-sterilization (0.2 µm). All cultures were grown in the dark at 23–25 °C*.*

**Table 1:** Composition of different media tested for induction of callus in *Stipagrostis pennata*

Name of medium	Base	BAP 0.4 mg·L^–1^	2,4-D 3 mg·L^–1^	Myo- Inositol 100 mg·L^−1^	Casein hydrolysate100 mg·L^−1^	Ascorbic acid 100 mg·L^–1^	CuSO_4_ 0.5 mg·L^–1^	Sucrose g·L^–1^	Maltose 10 g·L^–1^	l-Glutamine 500 mg·L^–1^	Gelrite 3 g·L^–1^
MS-T	MS	√	√	√	−	−	√	30	−	−	√
MS-Pw	MS	−	√	√	−	−	√	30	−	−	√
MS-Mod	MS	−	√	√	√	√	√	20	√	√	√
MS-FS	MS	−	√	√	√	√	√	20	√	√	√

MS-T (MS from stock solutions used in Teheran; autoclaved), MS-Pw (MS powder from Duchefa; autoclaved), MS-Mod (MS powder from Duchefa; autoclaved), MS-FS (MS powder from Duchefa; filter-sterilized)

### Plant regeneration and rooting

After eight weeks, embryogenic calli with a creamy color and globular surface were transferred (approximately 20 pieces of calli per plate) onto shoot induction medium MS including salts and vitamins, supplemented with 5 mg·L^–1^ zeatin riboside, 500 mg·L^–1^ L-glutamine, 100 mg·L^–1^ casein hydrolysate, 100 mg·L^–1^ ascorbic acid, 1.25 mg·L^–1^ CuSO_4_·5H_2_O and 3% sucrose. 3% gelrite was used for media solidification and pH was adjusted to 5.6–5.8. Cultures were grown for 2–3 weeks at 23–25 °C under continuous light at 40 µmol·m^–2^·s^–1^ in growth cabinet Percival (Percival Scientific, USA). Cultures were transferred to fresh medium every three weeks. When meristematic green zones appeared after the second sub-culture, cultures were transferred to fresh medium of the same composition and grown under light at 80 µmol·m^–2^·s^–1^ and a photoperiod of 16-h photoperiod (Grow Light Quattro, Venso EcoSolution, Finland). Well-developed elongated shoots were transferred to MS hormone-free medium, supplemented with 3% sucrose and grown for another 2–3 weeks at 23 °C under 16-h photoperiod under light at 80 µmol·m^–2^·s^–1^. Well-rooted plants were potted into Jiffy peat pots (Jiffy-7® Norway), placed in plastic containers with green filters (COMBINESS & Eco2NV, Belgium), and further grown in a controlled environment at 24 °C with a 16/8 h photoperiod for 5–6 weeks. When well-developed roots system had formed in the Jiffy pots, plants were transplanted to 1 L pots with regular greenhouse substrate and transferred to a greenhouse with controlled conditions (90% humidity, 24 °C, continuous light at 100 µmol·m^–2^·s^–1^).

### Data collection and statistical analyses

Callus induction, somatic embryo induction and regeneration of shoots were carried out in four different media (Table 1) used for testing four different types of initial explants: cut caryopsis, mature embryo, immature embryo and leaf base, from two geographical locations (Khuzentan and South Khorasan) in Iran. Sixty explants were used in each experiment. Each treatment consisted of three replications and the experiment was repeated three times.

Shoot induction for all the explants was recorded after maintaining the cultures for eight weeks at 80 µmol·m^–2^·s^–1^ and a photoperiod of 16-h. For statistical analyses, data were analysed by means of analyses of variance (ANOVA) using SAS (version 2.9). The treatments were grouped using the GLM Procedure (PROC GLM) method and analysed based on the Duncan’s Multiple Range Test for means comparison at 5% significance level. Graphs were plotted in Microsoft Excel.

### Histology

Samples for histology were collected from all explants 21–28 days after culture initiation on callus induction medium and 20–40 days after transfer to shoot induction medium. The tissues were fixed overnight in formalin/acetic acid/alcohol (FAA; 50% ethanol:10% formalin:glacial acetic acid, 18:1:1). Fixed tissues were dehydrated in ethanol and tertiary-butanol series, and embedded in LR White resin [63]. Serial sections were cut at 5 µm thickness on a rotatory microtome and stained with toluidine blue [64]. All sections were studied under light microscope (Axio Plan Imaging, Zeiss, Germany) and photographed with an attached camera (Axio Cam HRc, Zeiss, Germany).

### Somaclonal variation

Genome stability (or absence of somaclonal variation) was evaluated in three samples of in vitro regenerated shoots of *S. pennata* obtained from tissue culture and four seedlings derived from zygotic seed germination were used as controls. DNA from fresh, young leaves was extracted using E.Z.N.A.® Plant DNA DS Kit (Omega) following manufacturer’s instructions and DNA concentration was measured with Thermo 2000 Nanodrop. Ten pairs of SSR primers (Eurofins, Table 2) were selected based on the previous study on a closely related grass species (*Stipa pennata*) [65] as there is lack of any type of nucleotide sequence information available on the species of our interest. Polymerase Chain Reaction (PCR) was performed in 20 µL reaction mixture containing 50 ng genomic DNA, 0.5 µM of each primer (forward and reverse), 200 µM dNTP (Thermo Scientific™), 2.5 units DreamTaq DNA Polymerase (Thermo Scientific™), 1X PCR buffer (10X buffer with 20 mM MgCl_2_) and 1 µL Bovine Serum Albumin (BSA, 100 mg·mL^–1^ stock). PCR conditions for all primers pairs were as follows: initial denaturation at 95 °C for 3 min; 35 cycles of denaturation at 95 °C for 30 s, annealing at 53 °C for 30 s and extension at 72 °C for 30 s; final extension at 72 °C for 7 min PCR products were visualized on 3% agarose gel run with 0.5X TAE buffer, using 1 Kb DNA Ladder Plus (Thermo Scientific™) as marker.

**Table 2:** PCR primers used to analyze genomic stability of regenerated in vitro shoots obtained from embryogenic callus cultures of *Stipagrostis pennata*

Primer	Primer sequences (5′–3′)	Repeat motif	Allele size range	GenBank accession no
Primer1 (SP10)	F:CGCCTTTGTTGTTTATGAGCAG	(TA)_7_	165–185	MG978348
	R:AGCTAGTGTCCCACGTGTC			
Primer2 (SP12)	F:TAGATACGCCGGCTCGTT	GCCC)_4_	401–420	MG978349
	R:GTGATGGCAAGTACGGCAG			
Primer3 (SP41)	F:GGAAAGATGCGACAACCCG	(GAA)_4_	412	MG978355
	R:AACTTGAGCAGCCTCTTGG			
Primer4 (SP17)	F:ACTGTTGAAACCACGATCCG	(TAA)_4_	326–350	MG978351
	R:GCGGAACATTTGCCTTTGG			
Primer5 (SP43)	F:GGCAGAACAAATGGAGCCC	(AAT)_4_	323	MG978356
	R:GCAAACGCATCGAAACCTC			
Primer6 (SP23)	F:CTTAGCGCCTGGCCAAATC	(TA)_6_	297–309	MG978352
	R:CCTTTCCTGAAGCTAAACCGAC			
Primer7 (SP28)	F:AGGCTCAGTGTCCGCAGAAG	(TC)_6_	237–243	MG978353
	R:AGGCATAGCCAAATGCCAC			
Primer8 (SP30)	F:AAAGCGGACGGCATTGTTC	(TA)_7_	210	MG978354
	R:AGAAAGCAAGCTTACGGTGC			
Primer9 (SP08)	F:CCGGAAATACAATATCCTACCGC	(CAA)_3_	288–297	MG968959
	R:GTCCGGAGGTCTCTCAAGG			
Primer10 (SP15)	F:AGCGTAAAGCTCTCGAGTATG	(TTA)_4_	413–430	MG978350
	R:CGAAGGGAGTCGCAAATTCAC			

### Bacterial strains used for transformation

*Agrobacterium tumefaciens* strain LBA4404 harbouring a super binary plasmid was used for *S. pennata* transformation. This plasmid was carrying the *dsdA* encoding D-serine ammonia lyase, from *E. coli* [66] as a plant selectable marker gene and a *gus* (β-glucuronidase) reporter gene. *Agrobacterium* strain LBA4404 was prepared by inoculating a single colony from a freshly streaked LB plate in 20 mL of liquid LB medium [67]. The *A. tumefaciens* culture was supplemented with 50 µg·L^–1^ spectinomycin and 60 µg·L^–1^ rifampicin and was grown overnight in a rotatory shaker at 220–240 rpm in darkness at 28 °C for 16–18 h. After measuring, the optical density of the cultures (O.D. 0.5–0.6) the bacterial culture was centrifuged at 4000 rpm for 10 min and the supernatant was discarded. The bacterial pellet was then re-suspended in 20 mL of grass infection media [68] supplemented with 100 µM of acetosyringone and culture was then incubated at 120 rpm at 21 °C in darkness for 1.5 h.

### Tissue infection and GUS activity

For plant tissue transformation experiments, a 3–day–old suspension culture of *S. pennata *embryogenic callus (from all four expalnts) was moved into liquid culture supplemented with 100 µM of acetosyringone and mixed with liquid *Agrobacterium* strains LBA4404 carrying a super binary construct pSB111AGUSSXA. The *gus* and *dsdA* genes were driven by the maize ubiquitin 1 (Ubi-1) promoter. The co-culture of the tissue with bacteria was carried out in the dark at 21 °C [58]. After three days of co-cultivation, bacterial growth was stopped by pouring the suspension through a Büchner funnel and washed with autoclaved distilled water supplemented with 500 mg·L^–1^ cefotaxime. The callus cell suspension was plated on sterile filter paper and transferred onto the solid callus induction medium supplemented with 160 mg·L^–1^ ticarcillin disodium/clavulanate potassium. Plant tissue was collected in a small tube approximately ten days after transformation for analysis of *gus* gene expression [69].

## Results

### Embryogenic callus induction and plant regeneration

**Fig. 1:**
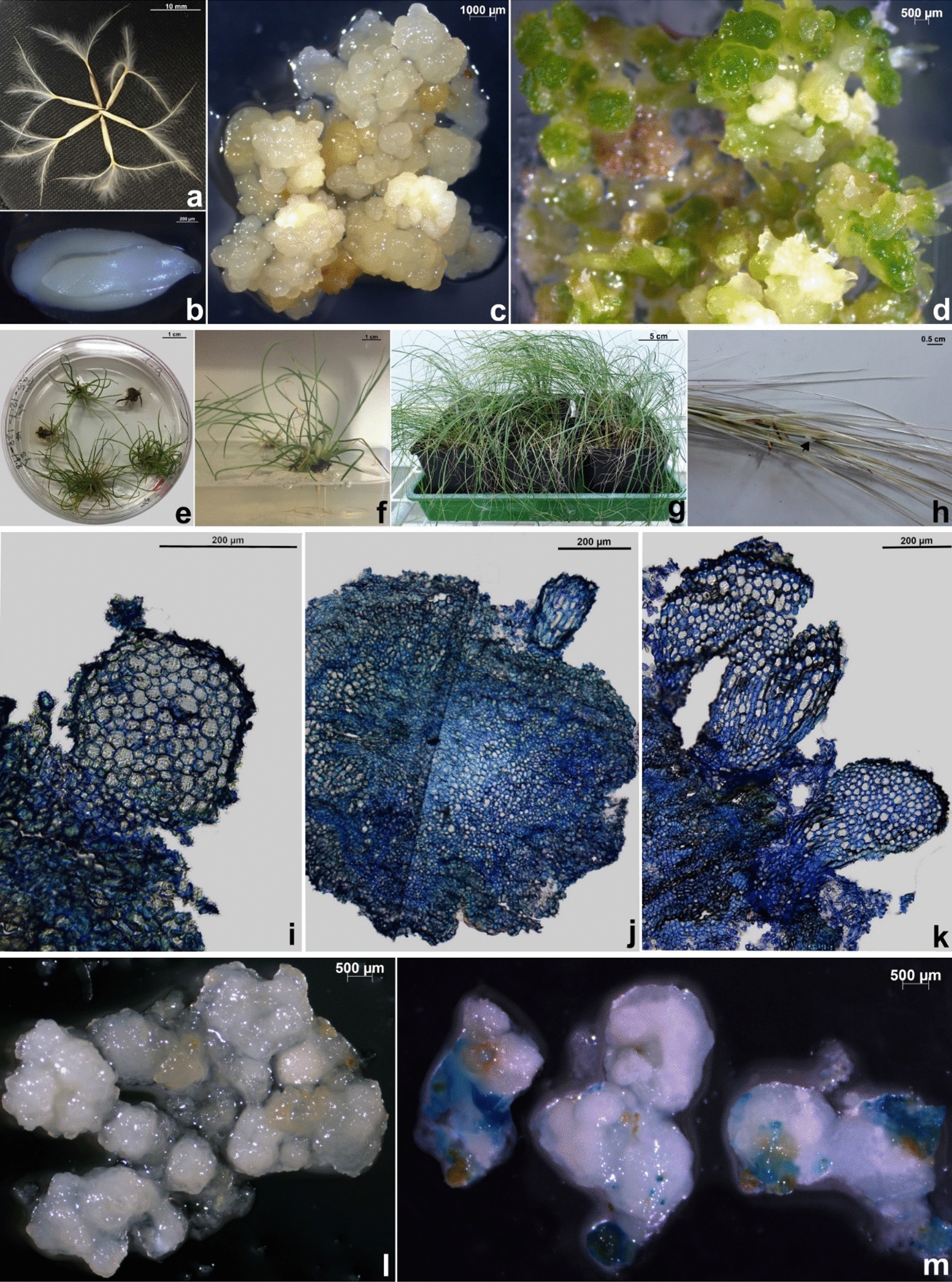
Somatic embryogenesis of *Stipagrostis pennata*: **a** mature seeds, **b** zygotic embryo, **c** induction of embryogenic callus, **d** induction of shoot on MS medium supplemented with zeatin riboside, **e** elongation of shoot on MS hormone free medium **f** rooted in vitro plants on MS hormone free medium, **g** plants adapted in greenhouse, **h **spike with the flower; arrow is showing anthers and stigma, **i **section of preglobular somatic embryo stage stained with toluidine blue, **j** section of globular embryo, **k** section of emryoids an advanced stage of somatic embryo **l** control embryogenic callus for *gus* gene expression, **m** *gus* gene expression in embryogenic callus

Embryogenic callus could be induced from all tested *S. pennata* explants (cut caryopsis, isolated immature zygotic embryos, mature zygotic embryos, and leaf bases) followed by shoots regeneration and transfer of rooted in vitro plants to greenhouse (Fig. 1a-h). Media tested were prepared in two different ways, by autoclaving or filter sterilization. The detailed composition of media used see in Table 1.

**Fig. 2:**
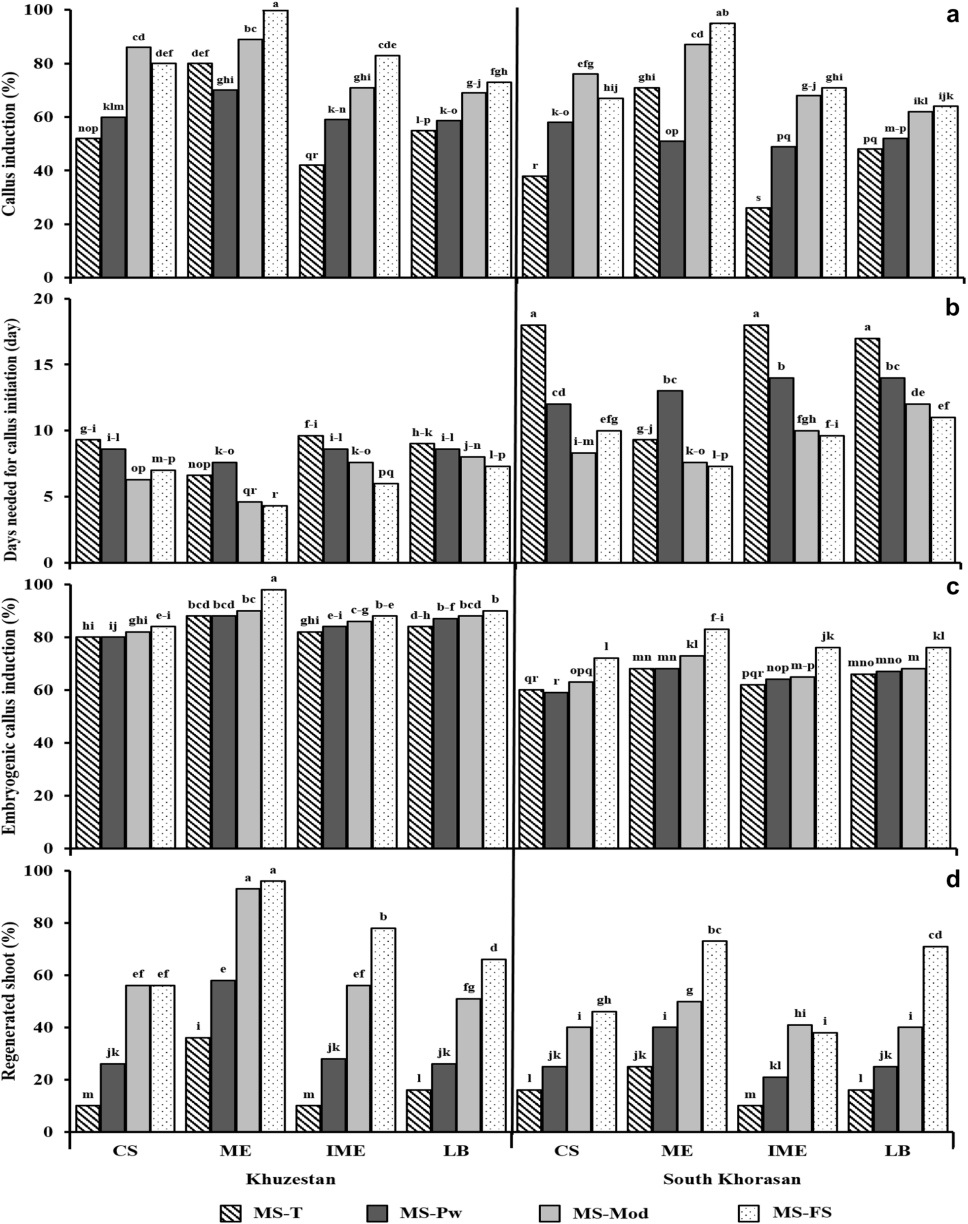
Responses from different initial explants of *Stipagrostis pennata* to embryogenic callus induction treatment***.* a** Rate of callus induction, **b** days on induction medium before callus emergence**, c** embryogenic callus induction, **d** regeneration of shoots from two geographical locations (Khuzentan and South Khorasan) in Iran in four different media used for testing with four different types of explants (CS: cut caryopsis, ME: mature embryo, IME: immature embryo and LB: leaf base). Duncan's Test categories are indicated on the top of the bars

Callus was induced from all four explants (cut caryopsis, isolated immature zygotic embryos, mature zygotic embryos, and leaf base) and appeared within one to two weeks. Callus was subcultured to fresh medium of the same composition every three weeks. Final scoring of callus induction was done after two sub-cultures to fresh medium and for the best responding explant type, mature embryos in combination with the filter-sterilized medium from both geographic locations, (Khuzestan 100% and South Korsahan 95%; (Fig. 2a) were the best responding explant type. When the speed of callus induction was compared between the two geographic locations, all four types of explants originating from South Korsahan (cut caryopses required 18 days, mature embryos 9.3, immature embryos 18 and leaf base 17 days) they needed almost two times longer period for callus induction as compared to the explants originating from Khuzestan (Fig. 2b). From this graph, it is clear that long induction time was required on medium MS-T for cut caryopses (9.3 days), immature embryos (9.6 days) and for the leaf base (9 days). Mature isolated zygotic embryos responded much faster on medium MS-T (6.6 days). The embryogenic callus induction from all four explants derived from South Khorasan and for all the four media that were used,

(MS-T 64%, MS-Pw 64%, MS-Mod 67% and MS-FS 77%) was lower in comparison to the ones derived from Khuzestan (MS-T 84%, MS-Pw 84%, and MS-Mod 86%; Fig. 2c). The highest embryogenic callus induction was observed when isolated mature zygotic embryos were grown on filter-sterilized (MS-FS 90%) medium supplemented with 2,4-D, Casein hydrolysate, L-Glutamine, Copper sulphate (CuSO_4_) and Ascorbic acid (Fig. 2c).

For shoot regeneration, MS filter-sterilized medium supplemented with 5 mg·L^–1^ zeatin riboside was used in all our experiments for all explants. Embryogenic callus from South Khorasan responded on this medium with the highest shoot induction from mature embryos (73%) and highest regeneration from leaf bases (71%; Fig. 2d). However, this response was still lower in comparison with the Khuzestan location. The highest shoot induction (96%) was obtained when embryogenic callus derived from mature embryos in combination with MS filter-sterilized medium was used or MS-Mod medium with 93% shoots induction respectively.

From all these comparisons, we can conclude that for the Khuzestan location, mature zygotic embryos as explant for embryogenic callus induction followed by shoot regeneration using the filter-sterilized media could be recommended as the best combination in our experiments.

## Histological observations

Mature and immature zygotic embryos approximately after 10–21 days in culture on callus induction medium started to produce compact and nodular callus, which is a typical characteristic for embryogenic callus in grasses. These nodular structures were often surrounded by a friable and translucent non-embryogenic callus. This stage of cultures with smooth, globular and pale yellow structures were fixed and embedded for the histological observation (Fig. 1c). Somatic embryos at different stage of development are visible in the sections stained with toluidine blue, proembryo structure (Fig. 1i) and globular embryos (Fig. 1 j) were visible. In the presence of high concentration of 2,4-D in the culture medium, the embryogenic nature of the callus can be maintained for some time. Frequent sub-culturing to the fresh medium (every two weeks) can help to continue with the embryogenic callus proliferation and development for up to two months (Fig. 1k). Once the embryogenic callus was transferred to shoot induction medium supplemented with 5 mg· L^–1^ zeatin riboside green, meristematic zone appeared in cultures and these structures were fixed and embedded in raisin for histological observations as well (Fig. 1d). When the cultures were transferred to medium with cytokinin in combination with the light culture conditions, then approximately 2–3 weeks later the green pockets in embryogenic callus were observed. Histological sections stained showed more advanced somatic embryos formation and developed of meristematic shoot buds (Fig. 1k).

### SSR marker analysis

**Fig. 3:**
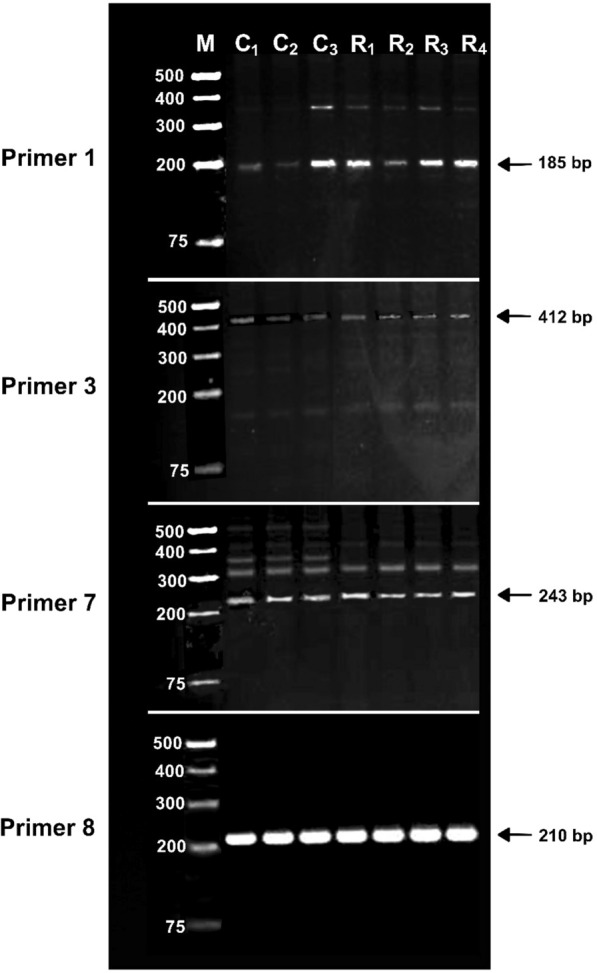
Agarose gel (3%) showing PCR amplification with the four SSR primers used for determining the genomic stability of the in vitro regenerated samples of *Stipagrostis pennata* (R_1_, R_2_, R_3_, R_4_). Seedlings of *S. pennata* from zygotic seed germination were used as controls (C_1_, C_2_, C_3_). DNA marker is indicated by M

For the current analysis, we tested 10 primer pairs (Table 2), out of which four primer pairs showed the expected size of amplification (Fig. 3): Primer1 showed a band size of 185 bp, Primer3 showed band size of 412 bp, Primer7 showed band size of 243 bp and Primer8 showed band size of 210 bp. The control and regenerated samples showed similar amplification pattern for all the four primer pairs, which confirms that somatic mutations were not detected in the regenerated samples for the loci that were tested. The PCR with the remaining primer pairs did not show expected amplicon sizes while non-specific amplification was observed in the control and regenerated samples.

### *Agrobacterium*-mediated transformation

Embryogenic calli derived from different explants: cut caryopses, mature and immature embryos, as well as calli obtained from the leaf segments were assayed for GUS activity. Non-transformed embryogenic calli derived from immature embryos were used as control (Fig. 1l) and here no blue staining was observed, confirming that there is no endogenous GUS activity in the tissue. In contrast, the positive *gus* reporter gene expression and blue coloration of tissue were obtained in transformed embryogenic calli derived from cut caryopses (Fig. 1m). The results confirmed that bacterial strain LBA4404 carrying a construct with *gus* reporter gene can be used for genetic transformation of *S. pennata* embryogenic callus.

## Discussion

### In vitro plants regeneration and histological observations

In grasses, the two major pathways for in vitro plant propagation through organogenesis and somatic embryogenesis were described in Gramineae already in the 80 s [70, 71]. Immature embryos are commonly used as an explant for somatic embryos induction of maize [72], rice [73], wheat [74], barley [68], and also for a model grass species [75, 76].

The use of mature embryos was reported by Luo, 2004 for bentgrass [77] and leaf base from *Panicum maximum* Jacq. by Lu and Vasil, 1981 [78], and for *Penisetum purpureum* Schum. by Haydu and Vasil, 1981 [79].

Our results indicated that mature embryos were suitable explant for callus induction with following embryogenic callus formation as well with the subsequent shoots regeneration. Mature embryos from the Khuzestan location reached 100% callus induction 90% embryogenic callus induction, and 96% shoots regeneration in combination with filter-sterilized medium.

Mature embryos from the other location, South Korsahan showed 95% callus induction, 77% embryogenic callus induction and only 73% shoots regeneration. We just can speculate that these differences are due to the different geographical locations, age of the seeds and e.g. the storage condition of mature seeds.

Cut caryopses, immature embryos, and leaf base (Fig. 2) can induce embryogenic callus and regenerate plants but with lower frequencies and also they need more days to start with callus induction.

Leaf explants started the callus induction on the cut ends where friable callus was formed and later on an embryogenic callus appeared after subculture to the fresh medium (data not showed).

Many years of work in plant tissue culture indicated that 2,4-D is a key growth regulator for inducing somatic embryogenesis in dicots [80], monocots [81], and trees [82]. When we used MS medium supplemented with 2,4 and BAP (Table 1; original MS Teheran medium) callus and embryogenic callus induction, and subsequent shoots regeneration for all explants tested from both locations was lower. This medium was sterilized by autoclaving what can affect the final quality of induction media [83] including pH what can have an impact on the cellular morphology of cultures and regeneration capacity as well [84, 85].

Shoot meristems are either developed or organized de novo in callus cultures [86] or are produced by derepression of existing meristematic shoot primordia in in vitro cultures which consists largely of proliferating meristems [87]. Shoot meristems both *ex vitro* and in vitro are considered as multicellular in origin, can produce chimeras [88] and the formation of shoot meristems from callus cultures typically results in higher level of cytological anomalies (Fig. 3).

The embryogenic callus as well as suspension cultures are genetically and cytological stable, and usually do not give rise to chimeric plants [81, 89]. This statement has also been confirmed in our work by analyzing SSR markers in the *S. pennata* plants regenerated in vitro from somatic embryos. For embryogenic callus induction with subsequent plant regeneration in monocotyledon species e.g. rice, isolated immature zygotic embryos are typically the explant of choice [73, 74, 90]. Identical explants were also used for *Brachypodium distachyon* L. by Pǎcurar et al. [75] whereas the use of mature zygotic embryos as initial explants was reported by Luo et al. [77] in bentgrass. Our histological sections of embryogenic callus showed early stages of somatic pro embryos and emryoids development similar to somatic embryogenesis reported by Lu and Vasil (1985) [70, 91] for *Panicum maximum ( guinea grass).*

Regenerated shoots on hormone-free MS medium easily formed roots and plants were successfully transferred to the soil wherein they were fully adapted to the controlled environment of the greenhouse in ex vivo condition, and they continued to grow.

### SSR marker analysis

Genome stability during somatic embryogenesis has been accessed by analyzing SSR markers e.g. in pine [92], spruce [93] and oak [94]. Assessment of genetic stability with SSR markers has been demonstrated in micro propagated plants species [95, 96]; particularly in grasses with economical value e.g. sugarcane [97]. Genetic fidelity in regenerated sugarcane through direct organogenesis was determined with SSR markers [98]. In the current work, SSR markers were assessed to determine the genetic stability of the regenerated plants. PCR amplification of the expected size was observed in four out of the ten SSR markers that were tested. Polymorphism was not observed in the regenerated *S. pennata* plants when compared to the controls, which suggests that in vitro procedures did not cause any mutations in the regenerated plants and the regenerated plants thus have a genomic stability. Although polyacrylamide gels and capillary electrophoresis using fluorescence-labeled SSR markers are the methods used to confirm the variations in the microsatellites, agarose gel electrophoresis is also used as a standard technique to confirm the polymorphisms in the SSRs which has been successfully applied in agricultural crops like sweet cherry [99], olive [100] and rice [101, 102].

Primer1 and Primer7 primarily amplifies the expected sizes, although there are some low amplifications of non-specific bands. These primers were originally designed for *Stipa* sp. which is a closely related species to *S. pennata* but yet a distinct species. Therefore, these primers seem to work for *S. pennata*, but also amplifies some other regions, which leads to higher band size. It is a worth to mention that there is lack of any type of nucleotide sequence information available for *S. pennata*, therefore primers designed for *Stipa sp.* were included for the current analysis.

The results from BLASTX performed with sequences of the PCR products of the respective primer pairs that showed the expected amplification size (GenBank accession numbers—Primer1: MG978348, Primer3: MG978355, Primer7: MG978353 and Primer8: MG978354) revealed that the Primer7 amplified sequence showed similarity with BSD domain-containing protein 1 gene. Primer1 and Primer8 PCR products showed similarity to hypothetical protein sequences. Thus three of the PCR products belonged to the coding region of the genome. Moreover, none of the regenerated samples showed somatic mutations in these coding sequences of the genome which suggests genomic stability in the coding parts for the loci analyzed. This reflects that the somatic embryogenesis procedures followed for the current work did not give rise to any somatic mutations for the loci tested and its worth mentioning that three of the loci tested belong to the coding region of the genome.

SSRs located in the coding regions are more relevant as compared to the ones that occur in the non-coding regions, primarily as the variations in the SSRs from coding regions would affect gene expression. Expressed sequence tags (ESTs) are potential candidates for development of genic SSR markers as well as applied for gene discovery, population genetic analysis and comparative genomic analysis [103]. Therefore, the EST-SSRs gained significant importance as “functional markers” that represent trueenetic diversity across the samples tested [104, 105]. Several studies have been carried out to assess the genetic diversity among different species as well as different cultivars or populations using EST-SSRs [106, 107]. Particularly in grasses, EST-SSRs were assessed for genetic diversity analysis across and within species e.g. in bamboo [29], sugarcane [108], forage grass species [109], switchgrass [110], napiergrass [111] and, various species of temperate forage and turf grasses [112]. Moreover, the non-coding SSRs are poorly conserved across species [113], which is also evident from our current work. Out of the 10 pairs of the primers tested, which belong to *Stipa pennata* species, four primer pairs showed the expected size amplifications in the current species of interest (*S. pennata*) and three of the primer amplicons were located in the coding regions of the genome.

### *Agrobacterium*-mediated transformation

Progress and challenges in *Agrobacterium*-mediated transformation in different grass species has been summarized by Giri and Praveena (2015) [114] and described in details for many grass species by different studies in bahiagrass (*Paspalum notatum*) [115, 116], rhodesgrass (*Chloris gayana*) [117], ruzigrass (*Brachiaria ruziziensis*) [118] and napiergrass (*Pennisetum purpureum* Schumach.) [76]. *Gus* (*uid*A) gene of *Escherichia coli* is the most widely used reporter gene to detect early steps of cell transformation*,* which was also successfully reported with reference to expression in turfgrass by Basu et al. (2004) [119] and by Luo et al. (2004) [77] in bentgrass. Our results, using embryogenic calli derived from cut caryopses of *S. pennata* testing GUS expression confirmed that “the blue gene” could be successfully used in this grass species as well. Similar results were obtained for callus induced from immature embryos of *Brachypodium distachion* [120], a model species for grasses.

## Conclusions

Our tissue culture protocol, which is developed and described here for the grass species *S. pennata,* includes embryogenic callus induction with genetically stable shoot regeneration, rooting in vitro*,* and successful adaptation of plants to ex vivo, in a greenhouse environment. This work, together with the positive expression of *gus* gene detected in the embryogenic calli, forms a solid base for the future transgenic plant production for the particular grass species which is of commercial value.

## Data Availability

All data generated or analyzed during this study are included in this published article.
